# Empowering Older Adults Through Values-Informed Solutions for Technology Adoption: Protocol for a Feasibility and Acceptability Randomized Controlled Pilot Trial

**DOI:** 10.2196/85257

**Published:** 2026-05-21

**Authors:** Melissa D Hladek, Olivia C Rubio, Samantha Curriero, Samantha Horn, Avrey Hughes, Deborah H Wilson, Mara McAdams-DeMarco, Deidra C Crews, Sarah L Szanton

**Affiliations:** 1 School of Nursing, Johns Hopkins University, 525 North Wolfe Street, Ste. N406, Baltimore, MD, 21205, United States, 1-(410) 614-2418; 2 Center on Aging and Health, Johns Hopkins University, Baltimore, MD, United States; 3 Ross and Carol Nese College of Nursing, Pennsylvania State University, State College, PA, United States; 4 School of Nursing, Auckland University of Technology, Auckland, New Zealand; 5 Department of Surgery, Langone Health and Grossman School of Medicine, New York University, New York, NY, United States; 6 Department of Population Health, Langone Health and Grossman School of Medicine, New York University, New York, NY, United States; 7 Division of Nephrology, School of Medicine, Johns Hopkins University, Baltimore, MD, United States

**Keywords:** self-efficacy, digital literacy, person-centered care, frailty, technology, in-home

## Abstract

**Background:**

Although technology usage is steadily increasing among older adults, adoption and confidence greatly lag behind their younger counterparts. Sociocultural and health disparities intersect with aging to present distinct structural and psychosocial barriers to the adoption of newer technologies. Digital health literacy interventions can improve task-specific skills, technological self-efficacy, and use frequency, but most do not systematically incorporate older adults’ values and goals, which are key drivers of sustained behavior change.

**Objective:**

The proposed study aims to develop and evaluate the acceptability and feasibility of a person-directed, values-based, in-home digital literacy intervention for older adults, entitled values-informed solutions for technology adoption (VISTA).

**Methods:**

VISTA begins with a values and goals discussion rather than a skills test, mapping “What Matters Most” to individualized, SMART (specific, measurable, achievable, relevant, and time-bound) technology goals. Over 8 to 12 weeks, interventionists co-developed personalized learning plans with participants, delivering up to 6 in-home biweekly visits and interim phone calls. The study provided a tablet and assistance with obtaining home internet when needed. Outcomes included digital literacy (Mobile Device Proficiency Questionnaire), technology and chronic disease self-efficacy, social networks, multimorbidity, and frailty (Fried Frailty Phenotype). Feasibility was assessed via recruitment, retention or completion, data collection rates, survey administration time, withdrawal, intervention fidelity, and per-person cost; acceptability was assessed via a postintervention satisfaction survey (Likert and open-ended items) and willingness to recommend.

**Results:**

Funding was secured in November 2023. Institutional review board approval, intervention development, and focus groups were completed throughout 2024. Recruitment and baseline assessments occurred from January 2025 to July 2025, enrolling 21 participants and randomizing 11 to immediate intervention and 10 to waitlist control (waitlist participants received the intervention after a 3-month control period). One consented participant was unable to participate early in the intervention and is not included in analyses. Inclusion criteria included being aged 65 years and older, having English proficiency, and demonstrating a willingness to improve digital literacy. Exclusion criteria involved severe cognitive impairment. At baseline, participants had a mean age of 75.7 (SD 7.74) years and were predominantly female (n=13, 65%) and Black (n=19, 95%); most reported having a low income (10/12, 83%), living alone (12/14, 85.7%), and multimorbidity (mean disease count 3.95, SD 2.46). Follow-up assessments concluded in March 2026; data cleaning and analysis are ongoing, with primary feasibility and acceptability findings anticipated for fall 2026.

**Conclusions:**

This protocol offers a unique model centering on the values and goals of older adults to improve access, use, and understanding of technology. Tapping into the motivators of older adults may provide a more beneficial way to encourage older adult technology use. VISTA could be useful in many general contexts, more specifically for older adults who are homebound or have serious illnesses, or as a preintervention for interventions involving advanced technology understanding.

## Introduction

### Background

As technology advances, it increasingly shapes the everyday lives of older adults [1]. Digital tools, such as remote patient monitoring, telemedicine, smartphone apps, and online support groups—are being actively investigated for their ability to deliver support across multiple domains of well-being and encourage aging in place [1-5]. When adopted, these technologies can help older adults access medical care from home, maintain social connections through video calls or messaging services, manage daily activities via smart home devices, and receive timely information about their health conditions. These solutions can enhance older adults’ independence, comfort, and safety, enabling them to remain living in their own homes for longer periods [6].

Digital literacy is the technical and cognitive ability to use communication technologies to find, evaluate, create, and communicate information [7]. Although technology usage is increasing among older adults, approximately 1 in 4 do not use the internet at all and less than half own a tablet [8]. These findings may be at least partially attributed to the fact that roughly 25% to 35% of US older adults lack home internet access [9]. Psychosocial barriers also disrupt older adults’ adoption and use of technology, including lack of awareness, low technological self-efficacy, low digital literacy, and negative attitudes toward technology [10-13]. In contrast, older adults with access to training and technical support are more likely to adopt technology [14,15]. Additionally, like their younger peers, older adults are more likely to adopt technology if they perceive it as valuable and useful for improving the quality of their lives [11,16].

Digital health literacy is considered a super determinant of health [17]. Sociocultural and health differences intersect with age in technology usage patterns. Physical frailty [18], lower income [19], and limited education are associated with reduced technology engagement and negative attitudes toward information and communication technologies (eg, tablets) [20]. High costs and limited access often prevent adoption, especially among culturally and linguistically diverse groups, who also encounter language and resource barriers [21,22], whereas White and English-speaking older adults use digital technology more frequently [23].

Growing evidence suggests that digital health literacy interventions may improve specific technology skills, increase technological self-efficacy, and enhance the frequency of technology use [24-28]. However, research objectives for these digital literacy interventions do not routinely consider participants’ goals and preferences, which we know are salient motivators to behavior change [29] and a focus of the age-friendly health system movement [16]. Digital literacy interventions often focus on a single technology or task (eg, electronic health record or portal tasks) [26,30] rather than developing transferable and individualized skills. Existing digital literacy programs also typically require that older adults go to a senior center, library, or access resources over the phone, thus introducing another barrier for older adults who are frail, functionally limited, and less socially active [31]. There are a few promising home-based programs such as Tech Allies [32], Talking Tech [33], and Project Wire Up [34], but these are mostly pilots or nonrandomized designs, and few combine individualized coaching, access provision, and explicit values-based tailoring in the same intervention.

### Background on VISTA Development

Values-informed solutions for technology adoption (VISTA) is a newly developed, person-directed, in-home digital skills intervention for older adults. It emerged from a 12-month human-centered design process (interviews and focus groups) in which older adults identified digital literacy—and the lack of home internet or devices—as barriers to health communication, information access, and social connection. VISTA’s design integrates three elements that address gaps in prior programs: (1) values-aligned goal setting (“What Matters Most” and SMART [specific, measurable, achievable, relevant, and time-bound] goals) to enhance perceived relevance and motivation; (2) graded, transferable skills training delivered at home using an age-friendly handbook and coached practice; and (3) access enablement through the provision of a tablet and assistance in obtaining affordable home internet, when needed. As such, VISTA is aligned with best practices in technology-based intervention protocol development [35]. Unlike many single-app or site-based trainings, VISTA pairs individualized coaching with barrier reduction (devices or connectivity) and explicitly links foundational digital skills to both digital health tasks (patient portals, telehealth, and information appraisal) and broader health-promoting uses (social connection, mobility, and community resources).

### Conceptual Framework

**Figure 1.**
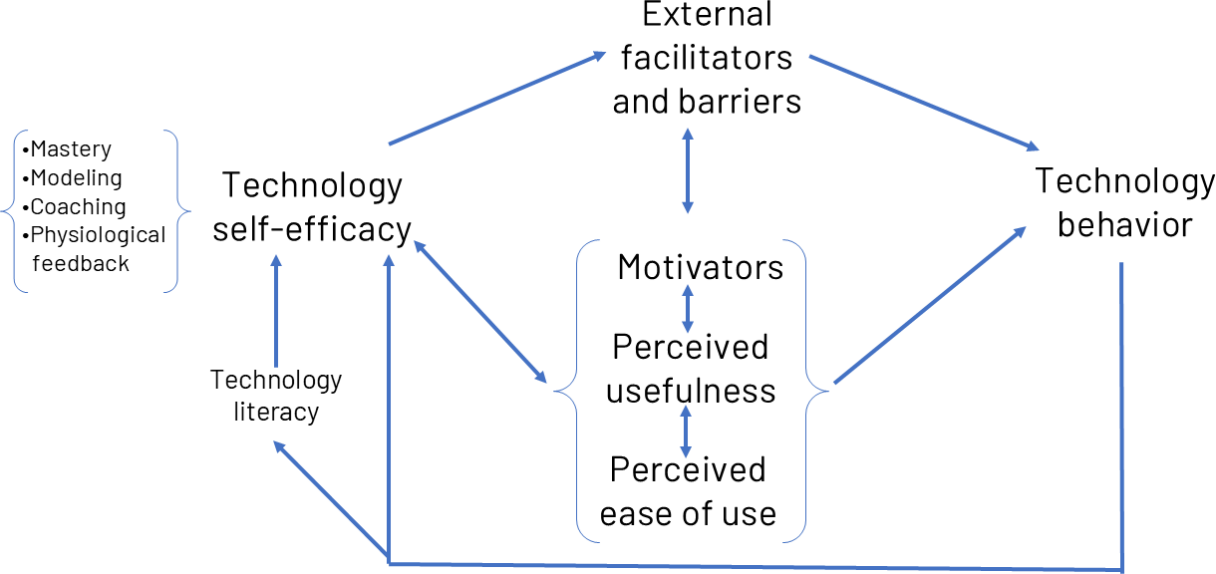
Values-informed solutions for technology adoption (VISTA) adapted conceptual model.

Our conceptual framework is adapted from 3 important theories of human behavior and digital uptake. Social cognitive theory, developed by Bandura [36], emphasizes the importance of observational learning, social influence, and the interaction between personal factors, behavior, and one’s environment (reciprocal determinism). Developed by the same psychologist, self-efficacy theory, which is the center of reciprocal determinism in social cognitive theory, refers to an individual’s confidence in their ability to perform specific tasks or behaviors and is associated with greater motivation and persistence in overcoming challenges [29,37]. The technology acceptance model describes how factors like the perceived usefulness and ease of use of technology influence one’s acceptance of that technology [38]. Our model (Figure 1) adaptation leverages all three theories to (1) build confidence in technology use through mastery experiences, modeling, social support, and positive reinforcement; (2) align technology use goals with what matters most to the older adult to enhance relevance and engagement; and (3) address technology preferences and reduce perceived barriers through internet access and user-friendly, age-appropriate training.

### Digital Health Literacy Versus Digital Literacy

Digital health literacy is typically defined as the ability to seek, find, understand, and appraise digital health information and apply this knowledge to address or solve a health problem [39]. In practice, for older adults, these capacities rest on broader, transferable digital literacy skills (eg, device navigation, communication, privacy or security management, and information appraisal) that enable both health-specific tasks (patient portal use, telemedicine, and secure messaging) and health-promoting activities beyond disease-specific clinical care (maintaining social connection via video calls, coordinating transportation to appointments, locating community resources, or identifying safe places to exercise). Consistent with social cognitive theory and self-efficacy theory, mastery of foundational digital skills and values-aligned, meaningful use can enhance perceived usefulness and confidence, increasing persistence and uptake of both health and health-adjacent digital behaviors.

Accordingly, our digital literacy intervention was designed to build transferable competencies and self-efficacy and to map them to individualized goals that include explicit digital health literacy tasks (eg, finding and interpreting health information, managing appointments or medications, using telehealth and portals) as well as health-promoting uses of the internet that influence quality of life and well-being (eg, social connectivity, mobility, and access to supportive services). We therefore conceptualize digital literacy and digital health literacy as overlapping, mutually reinforcing domains that together shape access to care, communication with clinicians, self-management, and risk factors such as loneliness and inactivity, which are key determinants of health in later life.

### Study Purpose

The proposed study aims to develop and evaluate the acceptability and feasibility of a person-directed, values-based digital literacy intervention designed for older adults with and without frailty in their homes. The study proposes to achieve this aim by recognizing and leveraging person-directed values and goals to drive motivation to learn new technological skills. Internet access and training are viewed as not only addressing health literacy and health system communication domains but also as (1) natural extensions of the home environment [40] and (2) methods to address the growing health disparities caused by unequal internet access and technology use [41,42].

## Methods

### Study Design

Guided by the conceptual framework outlined in the “Introduction” section, a single-blinded waitlist-controlled, randomized pilot trial was implemented to test the feasibility and acceptability of the VISTA intervention. The target sample size was 20. A sample size of 20 (10 per arm) consistent with CONSORT (Consolidated Standards of Reporting Trials) guidance (Checklist 1) for pilot or feasibility trials was selected to (1) evaluate recruitment, retention, protocol fidelity, and data collection burden; (2) obtain preliminary variance and effect-size estimates for powering a future trial; and (3) match operational capacity for delivering a resource-intensive, in-home intervention within the study timeline and budget [43]. Trial participants who enrolled were randomized into the immediate intervention or waitlist control groups. Randomization was implemented via the REDCap (Research Electronic Data Capture; Vanderbilt University) randomization module using a computer-generated sequence with concealed allocation, assigning participants in a 1:1 ratio to the immediate intervention or waitlist control arm [44]. The program coordinator then assigned participants accordingly and informed the intervention team of participants ready for the intervention. The principal investigator of the study remained blinded, and unblinding would only occur if essential for participant safety, institutional review board (IRB) reporting, or operational continuity; no unblinding was planned. The immediate intervention group (n=10) received the intervention during weeks 0 to 12. The waitlist control group (n=10) began the intervention approximately at week 12 through approximately week 24. A waitlist control design was used to provide an untreated comparison group while still allowing those participants to receive the intervention at a later date [45]. In-home research visits and survey instruments were collected at baseline and postintervention for the immediate intervention arm (T0 and T1), and at baseline, postcontrol or preintervention, and postintervention for the waitlist control arm (T0, T1, and T2). This randomized, waitlist-controlled pilot trial was registered at ClinicalTrials.gov (NCT06479707) in November 2024, protocol version v.1.0, prior to the start of recruitment; no protocol deviations were planned. This report adheres to the CONSORT 2010 extension for pilot and feasibility trials [43] and the TIDieR (Template for Intervention Description and Replication) checklist (Checklist 2) for intervention description [46]; protocol elements were informed by SPIRIT (Standard Protocol Items: Recommendations for Interventional Trials) 2025 [47] (Checklist 3). MDH is the principal investigator and overall guarantor of the protocol.

### Study Population and Recruitment

Recruitment took place through flyers distributed within the Baltimore community and surrounding counties in senior centers and senior living communities. Inclusion criteria included participants aged 65 years and older with English proficiency and a willingness to improve digital literacy. We excluded participants with severe cognitive impairment [48]. Participants randomized to the intervention completed a survey assessment during both a preintervention and postintervention visit with the research team. Additionally, control participants had one additional data collection visit postcontrol or preintervention. This minimal-risk behavioral pilot imposed no restrictions on concomitant care.

### Ethical Considerations

Prior to data collection, a research team member sat down with each participant to review the purpose of the study, the procedures during the study, confidentiality, and the risks and benefits of participation. They emphasized that participation in the study was voluntary and that participants could pause or withdraw from the study at any time. The research team member provided an opportunity for discussion and questions and assessed the participant’s comprehension of the information provided. Written consent was signed, with a copy given to the participant.

This study was approved by the Johns Hopkins University IRB (IRB00437921). Amendments will be approved by the IRB before implementation.

Data were stored on secure servers at Johns Hopkins University with firewalls and regular security monitoring. Access was role-based: interventionists accessed personally identifiable information required for delivering the intervention, while other study personnel accessed only deidentified data. All activity was logged via an audit trail, and data exports for analysis were deidentified whenever possible.

Given the minimal-risk nature of the intervention, adverse events were monitored by interventionists and reported to the principal investigator and IRB per institutional policy [49]; no external adjudication committee or data monitoring committee was convened. No interim analyses or formal stopping rules were planned.

Participants received a US $50 gift card during both the baseline and postintervention in-home survey visits.

### Intervention Development and Human-Centered Design

This intervention was theory-guided and developed, in part, using human-centered design principles. Human-centered design is a collaborative process involving the end user as part of the intervention development process in order to best understand and serve people’s needs [50]. Intervention development occurred over the course of 12 months and involved interviews and focus groups with older adults with multimorbidity, which identified digital literacy as a need to help facilitate overcoming many other health-related barriers around communication, health knowledge, and support [51].

### Intervention Components

Over the course of 8 to 12 weeks, participants received 4 to 6 in-home biweekly visits, along with 3 to 4 interim phone calls based on their individual digital literacy needs and goals. Each visit lasted approximately 1 hour. Each participant received a tablet if one was not already available. Participants without home internet were assisted in applying for low-cost internet services through federal, state, and local affordability programs, which were paid for by the study for the duration of the intervention. The difference in the number of visits was partially determined by the participant’s goals, current digital literacy, and existing internet connectivity. See Table 1 for more information on the intervention timeline and key components.

**Table 1.** Intervention visit timeline.

Time	Key activities
Baseline visit	Consent and baseline data collection
Week 1 (optional)	Granted for persons with no internet connectivity to aid in connection process
Week 2 (optional)	Granted for persons with no prior use of smart technology (phone or tablet) or who have never used a touchscreen before
Week 3	Eliciting general goals and values Applying to technology Cocreating technology-related SMART^a^ goals iPad set-up Unit 2 of handbook as related to goals Assign relevant activities
Week 4 (TC^b^)	Review progress, facilitators, barriers, and problem-solve
Week 5	Unit 3 and 4 as related to goals Assign relevant activities
Week 6 (TC)	Review progress, facilitators, barriers, and problem-solve
Week 7	Units 5, 6, and 7 as related to goals Assign relevant activities
Week 8 (TC)	Review progress, facilitators, barriers, and problem-solve
Week 9	Discuss goal attainment Recommend next steps
Postintervention visit	Postintervention data collection

a SMART: specific, measurable, achievable, relevant, and time-bound.

b TC: telephone call.

This pilot intervention centered on the participants’ own values and goals in order to co-develop biweekly individualized action plans with the interventionist. These action plans formed the basis of the intervention design. See examples of individualized action plans in Textbox 1. There were 4 main components to this intervention, namely (1) eliciting values and goals, (2) mapping values and goals onto technology goals, (3) co-creating action plans with participants using the goals worksheets, and (4) using the VISTA technology handbook to develop confidence and specific technological skills (Multimedia Appendix 1).

Textbox 1. **Examples of values-based technology goals and intervention approaches.**

The following are the example goals and goal-specific action plan items:

- Video conversations with grandson (action plan: talk to grandson on FaceTime calls 3 times weekly for 10-15 minutes).
- Access MyChart health record (action plan: practice signing in and navigating MyChart app to find recent laboratory results and visit notes).
- Manage my medications via Apple Health (action plan: navigate Apple Health app and enter current medication list).
- Connect with chronic disease support groups (action plan: practice navigating the internet and find at least 1 support group on a professional health organization website).
- Complete online age-appropriate exercise workout (action plan: use YouTube app to find Silver Sneakers videos and engage in 5-10 minutes of exercise 3 times weekly).

The following are the non–goal-specific action plan items (these action plan approaches are chosen by the interventionist and participant based on current digital literacy needs in order to achieve goal-specific action. This is not a comprehensive list of non–goal-specific action plan item options):

- Orient to the iPad home screen
- Practice different touchscreen gestures
- Learn how to charge my tablet
- Successfully connect iPad to WiFi
- Search the internet for organizations that advocate for people with my health condition
- Learn about tablet apps and how to download
- Successfully set up a social media account

The four main components of this intervention are described here.

**Figure 2.**
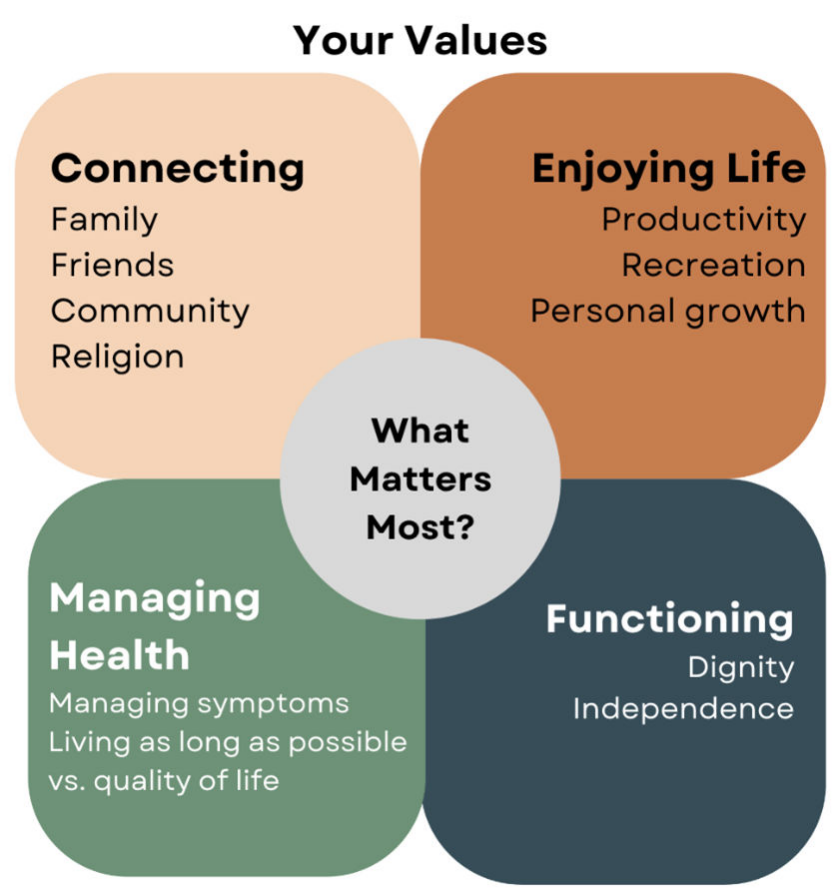
Identifying what matters most to the participant.

- Eliciting values and goals: the interventionist met with the participant to review their values and goals using the “What Matters Most” framework to guide the conversation [16,52,53]. This conversation started with questions like, “What does a good day look like for you?” Using Figure 2, the interventionist asked, “Which areas are particularly important to you?” This structured “What Matters Most” conversation identified priority domains (eg, health management, social connection, and daily functioning).
- Mapping values and goals onto technology: the interventionist and participant brainstormed different ways technology can be used to help the participant meet their goals. The interventionist asked, “How do you think the iPad may be able to help you live out your values?” They then engaged in an in-depth discussion about the participant’s past experiences using technology, including barriers, facilitators, and strategies to support learning and address barriers during the training.
- Action planning using goals worksheets: the interventionist provided a “What is a SMART Goal” handout and reviewed the information with the participant. Together, they worked on a “Technology SMART Goal Worksheet” to establish 1 to 3 technology goals for the intervention. SMART goals are clear and actionable objectives that are specific, measurable, achievable, relevant, and time bound [54]. The interventionist helped the participant reflect and write down their motivations for achieving the goal, potential obstacles, problem-solving strategies, and indicators that can be used to track progress toward achieving the goal.
- VISTA Handbook: a study handbook was developed to help facilitate basic technological knowledge through text and activities that can be assigned as part of individualized action plans. Based on the individualized goals co-created by the interventionist and participant, as well as baseline technological proficiency, specific activities and units within the VISTA Handbook were assigned to facilitate learning how to use technology and building confidence in using it (see Multimedia Appendix 1 for the “Table of Contents for the VISTA Handbook”). Participants worked through the seven modules within the handbook, covering: (1) device fundamentals and accessibility (navigation, keyboard, voice input, font or contrast, assistive touch, and apps); (2) communication and social connection (contacts, voice or video calls, SMS text messaging, and group chats); (3) information seeking and appraisal (browser use, search strategies, evaluating sources, and health information literacy basics); (4) health system navigation (patient portal account creation or recovery, secure messaging, viewing laboratory results or visit notes, appointment scheduling, telehealth setup, and etiquette); (5) organization and self-management (calendar, reminders or alarms, medication lists, photo or document capture, and storage); (6) internet and privacy (WiFi selection, hotspot basics when applicable, privacy settings, app updates, and permissions); and (7) problem solving (force-quitting, updates, storage management, and common error recovery).

Each biweekly visit included live demonstrations, participant practice, and brief at-home tasks aligned with SMART goals. Interim phone calls reinforced skills, addressed any problems or barriers that arose, and adapted the action plan. The interventionist adjusted the pace and content based on observed mastery and participant feedback, documenting modules completed with unit pretests and posttests, barriers encountered, and adaptations (eg, caregiver involvement and alternative apps with simpler interfaces).

These components operationalize the conceptual framework by building self-efficacy through graded mastery experiences and feedback (handbook modules and guided practice), enhancing perceived usefulness via explicit mapping of skills to personally meaningful goals, and reducing environmental barriers (device and internet access and in-home delivery).

### Interventionist Training

Interventionists were selected based on their expertise in technology, their experience working with older adults, and their skills in therapeutic communication and motivational interviewing. They received additional training in SMART goal setting, active listening, empathy, open-ended questioning, reflective responses, nonverbal communication, and the essentials of building rapport and trust. This training was accomplished through didactic sessions and role-playing scenarios, including assessing and working with participants at different stages of readiness for change [55]. Interventionists were directly supervised by the principal investigator and study coordinator, with regularly scheduled coaching throughout the intervention to refine skills and address challenges in difficult cases.

### Data Collection and Management

Study data were collected and managed using REDCap, a secure, web-based platform [44]. REDCap supports real-time data entry, validation checks, and branching logic to ensure data quality. The study team performed routine data quality checks, including range and logic checks, to ensure accuracy and completeness. Privacy and confidentiality protections are detailed in the “Ethical Considerations” section.

### Main Outcomes: Feasibility and Acceptability

Acceptability and feasibility were the main outcomes of interest in this pilot randomized controlled trial (RCT). Acceptability was assessed using a satisfaction survey at the end of the intervention, which included questions about the program content and structure, using Likert scale-based questions and open-ended questions, as well as participants’ willingness to recommend the intervention to others [56]. Feasibility was assessed by analyzing the following data: recruitment rate, completion rate, data collection rate, mean time for survey administration, withdrawal rate, and cost per person [43]. Attrition was defined as withdrawal from the study before completing the postintervention assessment (T1 for the immediate arm; T2 for the waitlist arm) and/or receipt of fewer than four in-home visits. Timing (prerandomization vs postrandomization; early vs late) and participant-reported reasons were recorded for discontinuation. Feasibility was also assessed through the evaluation of interventionist visit recordings and fidelity checklists. No changes to assessments or measurement procedures were made after trial commencement.

### Exploratory Outcomes and Covariates

Although not powered for effectiveness, our main exploratory outcome variable was digital literacy, collected using the 16-item Mobile Device Proficiency Questionnaire (MDPQ) [57]. The MDPQ has 8 subsections with 2 questions each on the following digital literacy topics: using a mobile device, communication, data and file storage, internet, calendar, entertainment, privacy, and troubleshooting and software management. The stem of each question starts with “Using a mobile device, I can…” and ends with statements like “setup a password to lock/unlock the device” or “find information about my hobbies and interests in the internet.” This scale measures all 5 competencies as outlined in the European Commission’s digital competence framework [58,59].

In addition to the MDPQ, we administered validated instruments capturing self-efficacy, stress exposure or appraisal, social engagement, shared decision-making communication, and frailty. Self-efficacy was assessed using 3 scales: the 13-item Coping Self-Efficacy Scale, which yields Problem Solving, Emotion Regulation, and Social Coping subscale scores with strong internal consistency (*α*=.91, .91, and .80, respectively) [60]; the 6-item Self-Efficacy to Manage Chronic Disease Scale (*α*=.88), capturing confidence in managing symptoms and health tasks [61]; and a 10-item Digital Self-Efficacy Scale (adapted from Ulfert-Blank and Schmidt; *α*=.96) indexing confidence in performing digital tasks [62]. Chronic stress was measured with a 24-item inventory combining stressor exposure counts and long- or short-term appraisal ratings (*α*=.75), encompassing general health, caregiving, physical and social function, financial, and emotional well-being domains [63,64]. Social engagement was assessed with the 6-item Lubben Social Network Scale (*α*=.83), which indexes perceived support and network size across family and friends [65,66]. Health team communication and shared decision-making were evaluated using the 3-item CollaboRATE measure (a fast, patient-reported assessment of shared decision-making; brief scale without internal consistency reporting) [67]. Frailty status was characterized with the 5-component Fried Frailty Phenotype (weight loss, exhaustion, low activity, slowness, and weakness), administered per standard protocols [68,69]. Covariates included age, sex, race or ethnicity, and chronic disease count. Table 2 outlines each construct and variable collected.

**Table 2.** Constructs, instruments, and reliability. Other variables collected include: age, sex, race/ethnicity, chronic diseases, and medications.

Theoretical construct, instruments, and variables	Number of items	Reliability (Cronbach α)	Assessment times
Digital literacy			
Mobile Device Proficiency Questionnaire [57]	16	0.99	T0, T1, T2^a^
Self-efficacy			
Coping Self-Efficacy Scale^b^ [60]	13	PS^c^: 0.91; ER^d^: 0.91; SC^e^: 0.80	T0, T1, T2^a^
Self-Efficacy of Chronic Disease Management [61]	6	0.88	T0, T1, T2^a^
Digital Self-Efficacy^f^ [62]	10	0.96	T0, T1, T2^a^
Chronic stress			
Stress exposure count and Long- or short-term stress appraisal^g^ [63,64]	29	0.75	T0, T1, T2^a^
Social engagement			
Lubben Social Network Scale [65,66]	6	0.83	T0, T1, T2^a^
Health team communication			
CollaboRATE Scale Measure [67]	3	—^h^	T0, T1, T2^a^
Frailty			
Fried Frailty Phenotype [69]	5	—	T0, T1, T2^a^

a T0: baseline; for the immediate intervention group: T1 was postintervention data collection; for the waitlist control group: T1 was postcontrol or preintervention data collection and T2 was postintervention. T2 data collection was only conducted on those randomized to the waitlist control group.

b Coping Self-efficacy Scale has 3 subscales: PS, ER, and SC.

c PS: Problem Solving Subscale.

d ER: Emotional Regulation Subscale.

e SC: Social Coping Subscale.

f This is an adapted version of the scale.

g Sections for general health, physical and social function, pain, and emotional well-being are included.

h Not applicable.

### Fidelity Plan

Our fidelity plan was modeled after the National Institute of Health Behavior Change Consortium Fidelity Plan, which was developed to ensure that behavioral interventions are implemented consistently [70]. Fidelity was maintained by (1) clearly defining the core components of the intervention, including specific content, delivery methods, and dose; (2) requiring consistent documentation, with 10% of sessions being audio-recorded and reviewed by the research coordinator using *a priori* fidelity checklists for monitoring content; (3) standardizing the training of our interventionists with ongoing assessment and coaching; and (4) assessing participant engagement.

Feasibility, acceptability, and fidelity data will be used to inform minor, process-focused refinements (eg, visit pacing, order of modules, reinforcement call cadence, troubleshooting scripts, and accessibility settings). Core components (values and goal elicitation, and SMART) action planning, VISTA Handbook content domains, and device or internet access enablement) will be maintained. All modifications and their rationale will be documented (TIDieR) and integrated into the standard operating procedures for a subsequent trial.

### Analysis

This is a pilot RCT primarily focused on feasibility and acceptability. Although not powered for effectiveness, descriptive analysis will be conducted for all study variables, scales, satisfaction, and feasibility data. Variables will be described using means and SDs or medians and IQRs accordingly. Regarding missingness, and given the pilot’s focus on feasibility and acceptability, the primary approach will be complete-case analysis with transparent reporting of missingness at each time point. Participants will also provide short-answer responses describing their personal goals and priorities related to technology. Using qualitative content analysis, responses will be coded into themes, enabling the team to explore patterns across participants and integrate these insights with outcomes from validated scales.

Effect sizes for digital literacy, the main exploratory outcome measure, will be assessed to determine the sample size needed to inform a larger future effectiveness trial. Between-group effect (primary exploratory contrast at T1): the authors will compute standardized mean differences for change scores (*Δ*=T1–T0) comparing the immediate intervention and waitlist control arms using Hedges *g* with small sample correction and 95% CIs. As a sensitivity analysis, the authors will estimate ANCOVA-based standardized mean differences by comparing T1 outcomes between groups while adjusting for T0 to improve precision. Within-group effects (pre-post change): the authors will estimate repeated-measures effect sizes within each arm using standardized mean change with the small-sample correction (repeated measures d, developed by Morris and DeShon; [71]) for T0→T1 in the immediate intervention arm and T1→T2 in the waitlist arm, with 95% CIs. Pre-post correlations will be estimated using observed data. Given the pilot nature and small sample size, hypothesis testing will be de-emphasized in favor of point estimates and CIs to inform sample size calculations and design parameters for a future trial.

## Results

Funding was secured in November 2023. IRB approval, intervention development, and focus groups all occurred throughout 2024. Participant recruitment and baseline assessments were conducted between January 2025 and July 2025, yielding 21 enrolled participants, 10 of whom were waitlist controls, receiving the intervention after a 3-month control period. One consented participant became medically unable to participate in the early stages of the intervention; results do not include this participant. Follow-up assessments concluded in March 2026, and data cleaning and analysis are ongoing, with primary feasibility and acceptability results anticipated for dissemination in Fall 2026. The baseline sample characteristics were as follows: mean age of 75.7 (SD 7.74) years, predominantly female (14/21, 85.7%), Black (20/21, 95.2%), low-income (10/12, 83.3%), and living alone (12/14, 85.7%) with multimorbidity (mean disease count 4.0, SD 2.46).

## Discussion

### Expected Findings

We find multiple strengths in this intervention. First, VISTA used a theory-based, person-directed, human-centered design approach. The intervention focuses on mapping participants’ own values and goals onto an action plan for building individualized digital skills that address their specific needs. In the current age-friendly health system movement, what matters most to the patient is becoming a more important metric to evaluate intervention and/or treatment effectiveness [72,73]. VISTA is person-directed and focuses on what matters most to the participant, aligning well with this emerging model of care for older adults. Additionally, by linking foundational digital skills to health-specific tasks (patient portals, telehealth, and secure messaging) and to health-promoting activities (social connection, appointment or medication management, transportation, and resource navigation) as directed by the participant, VISTA is designed to improve access to care, quality of clinician-patient communication, and self-management while mitigating risk factors such as loneliness and inactivity that adversely affect health in later life.

Designing VISTA to be person-directed also improves health fairness and parity. A one-size-fits-all intervention design does not account for the diversity of goals and values nor the potential needs of each person, which aligns with current thinking [74].

Additionally, VISTA focuses on improving digital equity for vulnerable older adults by reaching older adults with functional limitations within the home. The intervention also offers assistance with securing home internet and supplying technology. Both of these features of VISTA remove barriers to accessing technology, such as lack of transportation, limited mobility, and cost concerns. Delivering the intervention within the home environment may also reduce anxiety, leading to better overall engagement and learning outcomes. It provides insight into how older adults’ environments influence the adoption of technology, their experiences as end users of digital technology, and their unique preferences and values when it comes to engaging with technology.

### Limitations

The study recruited a small sample of 20 participants, which limits the generalizability of the study findings. Recruiting older adults aged 65 years or older who are proficient in English and without severe cognitive impairment, predominantly located in the Baltimore region, further restricts its generalizability. Requiring a “willingness to improve digital literacy” may also introduce selection bias toward motivated participants. This requirement likely enriched the sample with individuals who had greater baseline motivation and openness to technology, which could inflate feasibility, acceptability, and proximal skill gains relative to an unselected population that includes individuals who are ambivalent or resistant to technology. Although we intentionally reduced structural barriers by providing devices and internet access, delivering training in the home, and enrolling older adults with frailty and low income, the observed engagement and early outcomes should be interpreted as estimates among motivated users and may not generalize to all older adults. However, the purpose of the study was simply to test whether the intervention is feasible, to see if participants remain engaged, and to gather their feedback. This intervention may not address the needs of culturally or linguistically diverse populations that experience additional barriers to accessing technology, such as geographic isolation and language or communication challenges.

Despite study limitations, this protocol offers an innovative model for helping vulnerable older adult populations improve access to technology and understand the benefits it can offer using their own values and goals as a guide. Future preliminary expansion plans include incorporating multilingual facilitators to reach older adults with language barriers, considering adaptations for use in a rural context, using it as a “pretech intervention” to address recruitment bias in technology intervention studies and resulting health inequities, and applying it in other settings such as assisted living and skilled nursing communities.

### Dissemination

We aim to disseminate our findings at national conferences to gather feedback from experts in the fields of aging and technology. The study findings will be published in a reputable peer-reviewed journal and will be shared in plain-language with participants and community partners. ClinicalTrials.gov will also be updated with results upon completion. Furthermore, the results of this feasibility and acceptability study will be used to inform process refinements within the design of an RCT to evaluate the efficacy and effectiveness of the intervention. Future research will assess the cost versus benefit of the intervention and devise strategies for long-term sustainability and adaptation for diverse contexts.

## Supplementary material

10.2196/85257 Multimedia Appendix 1 VISTA handbook table of contents.

10.2196/85257 Checklist 1 CONSORT 2010 checklist.

10.2196/85257 Checklist 2 TIDieR checklist.

10.2196/85257 Checklist 3 SPIRIT checklist.

## Abbreviations

CONSORT: Consolidated Standards of Reporting Trials
IRB: institutional review board
MDPQ: Mobile Device Proficiency Questionnaire
RCT: randomized controlled trial
REDCap: Research Electronic Data Capture
SMART: specific, measurable, achievable, relevant, and time-bound
SPIRIT: Standard Protocol Items: Recommendations for Interventional Trials
TIDieR: Template for Intervention Description and Replication
VISTA: values-informed solutions for technology adoption
