# Supplementary material for: Empowering Older Adults Through Values-Informed Solutions for Technology Adoption: Protocol for a Feasibility and Acceptability Randomized Controlled Pilot Trial
Source: JMIR Res Protoc. 2026 May 21;15:e85257. doi: 10.2196/85257 (PMC13193668; doi:10.2196/85257)
Supplement: Multimedia Appendix 1 [file resprot-v15-e85257-s001.docx]

**Supplemental Table 1**

**Table of Contents for VISTA Handbook**


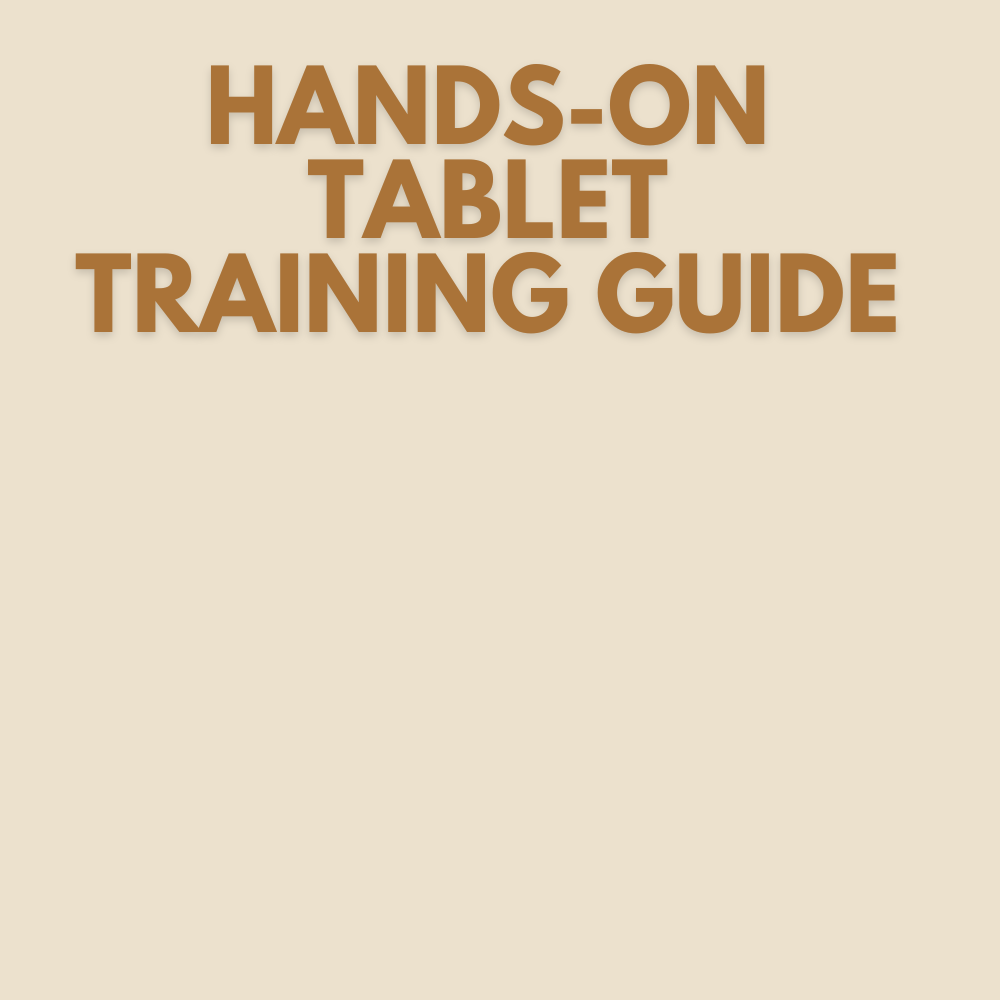

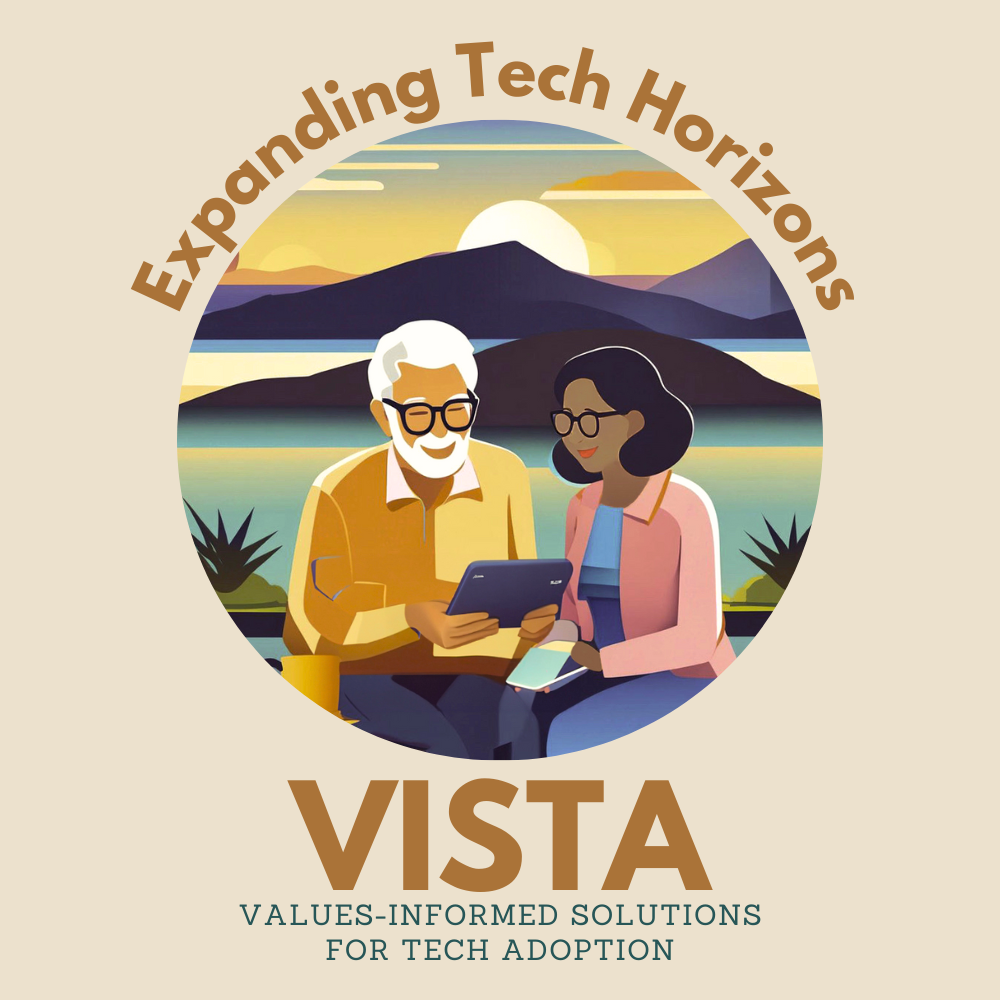
**“Hands-On” Tablet Training for Older Adults**

**Table of Contents**

[**Acknowledgements** 8](#_Toc175827205)

[**Preface** 9](#_Toc175827206)

[**Unit One: Introduction** 13](#_Toc175827207)

[Activity #1: Brainstorm Tablet Use 15](#_Toc175827208)

[Activity #2: Your Guiding Values 16](#_Toc175827209)

[What’s a Tablet? 18](#_Toc175827210)

[Activity #3: Past Learning & Technology Experiences 20](#_Toc175827211)

[Benefits of Technology to Older Adults 23](#_Toc175827212)

[The iPad 25](#_Toc175827213)

[Key Features of Your iPad 27](#_Toc175827214)

[Activity #4: iPad Features 28](#_Toc175827215)

[What is a S.M.A.R.T. Goal? 29](#_Toc175827216)

[Technology S.M.A.R.T. Goal Worksheet 30](#_Toc175827217)

[**Unit Two: The Basics** 33](#_Toc175827218)

[The Basics: Pre-Test 34](#_Toc175827219)

[Step-By-Step Process to Set-Up Your iPad: 37](#_Toc175827220)

[Locking and Unlocking your iPad 43](#_Toc175827221)

[Activity #5: Basic Features of Your iPad 44](#_Toc175827222)

[What you see – Your Home screen 45](#_Toc175827223)

[Getting Oriented with the Home Screen 47](#_Toc175827224)

[Activity #6: The iPad “Home” Screen 49](#_Toc175827225)

[Basic gestures 50](#_Toc175827226)

[Activity #7: Using Your Finger on the iPad (Gestures) 53](#_Toc175827227)

[Activity #8: Practicing Gestures 54](#_Toc175827228)

[Screen rotation 55](#_Toc175827229)

[Charging Your iPad 56](#_Toc175827230)

[Close Apps and Turn off your iPad 59](#_Toc175827231)

[Activity #9: Learning New Words about the iPad 60](#_Toc175827232)

[Charging a Tablet 60](#_Toc175827233)

[Locking your device 61](#_Toc175827234)

[Steps to set up Touch ID: 61](#_Toc175827235)

[Steps to set up a screen lock 62](#_Toc175827236)

[Security 62](#_Toc175827237)

[Changing Settings 64](#_Toc175827238)

[Font Sizes 64](#_Toc175827239)

[Activity #10: Adjusting Font Size 65](#_Toc175827240)

[Activity #11: Changing the Wallpaper (Background) 67](#_Toc175827241)

[Typing on the keyboard 68](#_Toc175827242)

[Using Siri 68](#_Toc175827243)

[Activity #12: Practice Using Siri (Voice Commands) 70](#_Toc175827244)

[Unit Two: The Basics: Post-Test 71](#_Toc175827245)

[**Unit Three: Getting Online** 73](#_Toc175827246)

[Unit Three: Getting Online: Pre-Test 74](#_Toc175827247)

[Unit Three: Getting Online 75](#_Toc175827248)

[Wi-Fi security 76](#_Toc175827249)

[Connecting to Public Wi-Fi 77](#_Toc175827250)

[How to Connect to a Wi-Fi Network 78](#_Toc175827251)

[Activity #13: Connecting to Wi-Fi 80](#_Toc175827252)

[Activity #14: Wi-Fi Settings Review 81](#_Toc175827253)

[Activity #15: Using the Internet 82](#_Toc175827254)

[Using the Internet 82](#_Toc175827255)

[Activity #16: Reviewing Steps to Using the Internet 86](#_Toc175827256)

[Using the Internet 87](#_Toc175827257)

[Activity #17: Searching the Internet 89](#_Toc175827258)

[Unit Three: Getting Online: Post-Test 90](#_Toc175827259)

[**Unit Four: Apps** 91](#_Toc175827260)

[Unit Four: Apps: Pre-Test 92](#_Toc175827261)

[Unit Four: Introduction to Apps 93](#_Toc175827262)

[Activity #18: Apps Matching Exercise 94](#_Toc175827263)

[Finding a specific app 95](#_Toc175827264)

[To add/move an app on the Home screen 96](#_Toc175827265)

[Removing App shortcuts from the Home Screen 97](#_Toc175827266)

[Activity #19: Moving Apps to the Home Screen 97](#_Toc175827267)

[Apps that come pre-loaded on your Tablet 97](#_Toc175827268)

[Specific Apps: Clock 98](#_Toc175827269)

[Activity #20: Set An Alarm Using the Clock App 100](#_Toc175827270)

[Specific Apps: Camera 101](#_Toc175827271)

[How to Use the Camera App 102](#_Toc175827272)

[Activity #21: Using the Camera App 104](#_Toc175827273)

[Specific Apps: Weather 105](#_Toc175827274)

[Specific Apps: Maps 106](#_Toc175827275)

[Activity #22: Using the Map App 107](#_Toc175827276)

[Adding Apps to the Tablet 108](#_Toc175827277)

[How to find an app 108](#_Toc175827278)

[Steps to Download an App 110](#_Toc175827279)

[App safety 111](#_Toc175827280)

[How to delete/uninstall an app 112](#_Toc175827281)

[Activity #23: Downloading & Deleting an App 113](#_Toc175827282)

[How to Download and Delete an App 114](#_Toc175827283)

[Additional Apps that may be of interest to Older Adults 116](#_Toc175827284)

[Activity #24: Setting up a New App 118](#_Toc175827285)

[Unit Four: Apps: Post-Test 119](#_Toc175827286)

[**Unit Five: Communication and Social Media** 121](#_Toc175827287)

[Unit Five: Communication and Social Media: Pre-Test 122](#_Toc175827288)

[Email 123](#_Toc175827289)

[Setting Up Your Accounts - Gmail 124](#_Toc175827290)

[Other Email Accounts 125](#_Toc175827291)

[How to Set Up an Email Account 126](#_Toc175827292)

[Composing and Sending Gmail 127](#_Toc175827293)

[Activity #25: Sending E-Mail with Gmail 128](#_Toc175827294)

[Contacts 129](#_Toc175827295)

[FaceTime 131](#_Toc175827296)

[Activity #26: Using Facetime 132](#_Toc175827297)

[Facebook 133](#_Toc175827298)

[Activity #27: Using Facebook 134](#_Toc175827299)

[Unit Five: Communication and Social Media: Post-Test 135](#_Toc175827300)

[**Unit Six: Navigating the Apple Health App** 137](#_Toc175827301)

[Unit Six: Navigating the Apple Health App: Pre-Test 138](#_Toc175827302)

[Apple Health App: 139](#_Toc175827303)

[How to set up an Apple Health Profile: 140](#_Toc175827304)

[Activity #28: Setting up your Apple Health Profile 141](#_Toc175827305)

[Navigating the Apple Health App 142](#_Toc175827306)

[Choosing your own health categories to track: 142](#_Toc175827307)

[Know how your health changes over time 143](#_Toc175827308)

[Choose which apps share information with Health 144](#_Toc175827309)

[Activity #29: Set Up and Track Your Health 145](#_Toc175827310)

[Activity #30: Setting up your Apple Health Profile 148](#_Toc175827311)

[Unit Six: Navigating the Apple Health App: Post-Test 149](#_Toc175827312)

[**Unit Seven: Navigating MyChart** 151](#_Toc175827313)

[Unit Seven: Navigating MyChart App: Pre-Test 152](#_Toc175827314)

[MyChart App: 153](#_Toc175827315)

[Creating a MyChart Account: 154](#_Toc175827316)

[Finding Upcoming Visits or Scheduling a Visit in MyChart 155](#_Toc175827317)

[Activity #31: Practice Scheduling Visits in MyChart 157](#_Toc175827318)

[Locating Your Test Results on MyChart: 158](#_Toc175827319)

[Reviewing Past Office Visit Notes 159](#_Toc175827320)

[Activity #32: Practice Finding Your Test Results 162](#_Toc175827321)

[Unit Seven: Navigating the MyChart App: Post-Test 163](#_Toc175827322)

[**Definitions/Glossary** 165](#_Toc175827323)

[Definitions/Glossary 166](#_Toc175827324)

[**Appendix** 169](#_Toc175827325)

[Appendix: “Hands-On” Tablet Training Activities 170](#_Toc175827326)

[Appendix: Technology S.M.A.R.T. Goal Worksheets – Extra Copies 173](#_Toc175827327)
